# Identification of a Genomic Region Containing a Novel Promoter Resistant to Glucose Repression and Over-Expression of β-Glucosidase Gene in *Hypocrea orientalis* EU7-22

**DOI:** 10.3390/ijms14048479

**Published:** 2013-04-17

**Authors:** Chuannan Long, Yijin Cheng, Lihui Gan, Jian Liu, Minnan Long

**Affiliations:** School of Energy Research, Xiamen University, Xiamen 361102, China; E-Mails: longcn2004@126.com (C.L.); chengyijinshin@126.com (Y.C.); laogan12@163.com (L.G.); jianliu@xmu.edu.cn (J.L.)

**Keywords:** *Hypocrea orientalis* EU7-22, promoter *proA*, β-glucosidase gene, over-expression, glucose repression, enzyme activity

## Abstract

A high concentration of glucose in the medium could greatly inhibit the expression of cellulase in filamentous fungi. The aspartic protease from fungus *Hypocrea orientalis* EU7-22 could efficiently express under both induction condition and glucose repression condition. Based on the sequence of structure gene of aspartic protease, the upstream sequence harboring the putative promoter *proA* for driving the expression of aspartic protease was obtained by genome walking. The upstream sequence contained the typical promoter motifs “TATA” and “CAAT”. The β-glucosidase gene (*Bgl1*) from *H. orientalis* was cloned and recombined with promoter *proA* and terminator *trpC*. The expression cassette was ligated to the binary vector to form pUR5750-Bgl1, and then transferred into the host strain EU7-22 via *Agrobacterium tumefaciens* mediated transformation (ATMT), using hygromycin B resistance gene as the screening marker. Four transformants Bgl-1, Bgl-2, Bgl-3 and Bgl-4 were screened. Compared with the host strain EU7-22, the enzyme activities of filter paper (FPA) and β-glucosidase (BG) of transformant Bgl-2 increased by 10.6% and 19.1% under induction condition, respectively. The FPA and BG activities were enhanced by 22.2% and 700% under 2% glucose repression condition, respectively, compared with the host strain. The results showed that the putative promoter *proA* has successfully driven the over-expression of *Bgl1* gene in *H. orientalis* under glucose repression condition.

## 1. Introduction

Lignocellulosic biomass represents abundant polysaccharides on the earth and a renewable resource, which can be used to produce environment-friendly biofuels, chemicals, polymers and materials [1]. Conversion of lignocellulosic biomass into fermentable sugars mainly depends on the degradation of cellulolytic enzymes produced by many filamentous fungi, including *Trichoderma*, *Aspergillus*, *Penicillium* and *Acremonium*[2,3]. The cellulolytic enzyme complex consists of three basic categories of enzymes: cellobiohydrolases (EC 3.2.1.91), endoglucanases (EC 3.2.1.4) and β-glucosidases (EC3.2.1.21), which act synergistically to degrade insoluble cellulose to glucose [4]. However, when filamentous fungi yielded large amounts of biomass-degrade enzymes protein, it was regulated by carbon catabolite repression (CCR), which mainly controls carbon assimilation [5]. The C2H2 type transcription factor CreI/CreA, has been shown to act as a repressor mediating CCR, which binds to the promoter of target genes *via* the consensus motif 5′-SYGGRG-3′, whose function *in vivo* has been shown in both *Trichoderma reesei*[5,6] and *Aspergillus nidulans*[7]. Cellulase production could be induced by cellulose, but strongly repressed by the major end-product hydrolysate-glucose.

For efficient expression gene in filamentous fungi, it is essential that the expression of the target gene would be enhanced by use of an active promoter either derived from the host or a related species. The cellobiohydrolase I gene (*cbh1*) promoter from *T. reesei* has been considered the strongest promoter, and has generally been used to construct high-efficient expression vectors to yield homologous and heterologous proteins [8]. However, the *cbh1* promoter is repressed by glucose and negatively regulated by Cre I/CreA [5,9]. Therefore, *cbh*1 promoter couldn’t express the target gene efficiently in the high concentration of glucose-containing medium. Bando *et al.*[10] found a novel promoter from a hemolysin-like gene (*hlyA*), which was not repressed by glucose and could efficiently over-express protein in *Aspergillus oryzae* grown in solid-state culture.

In the present study, a novel putative promoter *proA* regulating the expression of aspartic protease gene was isolated from *Hypocrea orientalis* EU7-22 by genome walking technology. The over-expression of β-glucosidases gene (*Bgl1*) in strain EU7-22 driven by putative promoter *proA* was studied.

## 2. Results and Discussion

### 2.1. Promoter proA Cloning and Sequence Analysis

**Figure 1:**
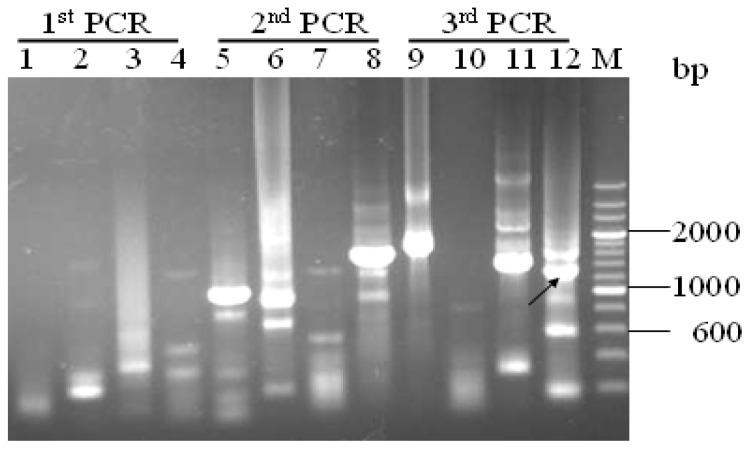
Gel electrophoresis of amplified products after the three nested genome walking PCRs. Lanes 1–4 were the first nested PCR products with the genome DNA as template using primer SP1 and AP1, SP1 andAP2, SP1 and AP3, SP1 and AP4, respectively; Lanes 5–8 were the second nested PCR products with the firstly nested PCR products as template using primer SP2 and AP1, SP2 and AP2, SP2 and AP3, SP2 and AP4, respectively; Lanes 9–12 were the third nested PCR products with the secondly nested PCR products as template using primer SP3 and AP1, SP3 and AP2, SP3 and AP3, SP3 and AP4, respectively. The arrow indicates the target amplified fragment of the unknown region. Lane M represents the DNA size standard markers (200 bp DNA Ladder Marker). The numbers on the right represent the DNA fragment length in bp.

The extension-based genome walking (E-GW) method was used to amplify the unknown 5′-upstream sequences of the *proA* gene. The three nested PCR products were analyzed by agarose gel electrophoresis (Figure 1). Following the Genome Walking Kit instructions, the DNA fragment (approximately 1200 bp) was obtained (Figure 1, lane 12, indicated by the arrow) with the primer AP4 (supplied by the kit) and reverse primers SP3, which was excised, purified and directly sequenced.

**Figure 2:**
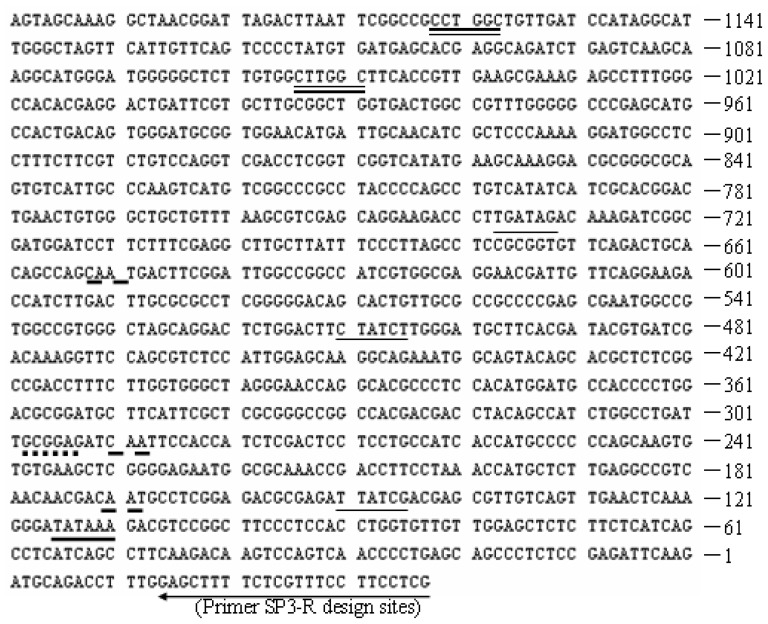
The nucleotide sequence of upstream of *proA* gene. The putative TATA (Indicated by “**——**”) and CAAT (Indicated by “**– –**”) boxes are indicated. The consensus binding sequences for the fungal transcription factors CRE I (5′-SYGGRG-3′, indicated by “**······**”), AREA (5′-HGATAR-3′, indicated by “**——**”), and PACC (5′-GCCARG-3′, indicated by “ 
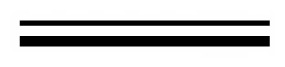
”) involved in carbon, nitrogen, and pH regulations, respectively. Arrow underline the sequences indicated the reverse primer SP3. M = A or C; S = G or C; Y = C or T; R = A or G; H = A, C or T.

The upstream sequences of *proA* gene were successfully cloned and deposited at the GenBank database with accession no. JQ728540. Homology analysis with NCBI Blast search showed 91% nucleotide identity to the *proA* gene upstream sequence (only 438 bp in length, from the start codon ATG) from *Hypocrea jecorina* (GenBank accession No. AM168137.1). A putative TATA box (TATAAA) was found at position −116 (relative to the ATG), and three putative CAAT boxes appeared at positions −172, −291 and −653 (Figure 2). Both motifs are related with the initiation of transcription [11]. The sequence contained multiple copies of the 5′-HGATAR-3′ motif, which corresponds to the nitrogen regulator AreA [12,13], located at −151, −511 (negative strand), and −738. Two 5′-GCCARG-3′ motifs for the PacC protein, which mediates pH regulation in *Aspergillus*[11], were also present at positions −1055 and −1163 (negative strand). A single copy of the motif 5′-SYGGRG-3′, the consensus sequence for the carbon catabolite repressor *Cre* I [5,9], was located at position −299. This was different from the promoter of *papA* gege encoding for aspartic protease from *Trichoderma harzianum* CECT 2413 which lacked potential *Cre*A sites for carbon regulation [14]. The secreted protein aspartic protease was strongly expressed even under the presence of 3% glucose in the medium (data not shown). It suggested that the putative promoter *proA* was resistant to glucose repression, which could be applied for constructing high-efficiency cellulase expression system.

### 2.2. Over-Expression of β-Glucosidase Gene in *H. orientalis*

**Figure 3:**
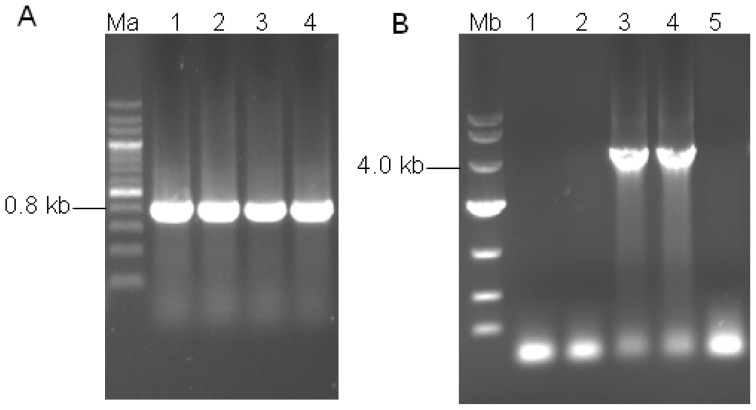
Molecular analysis of integration of T-DNA into *H.orientalis*. (**A**) PCR analysis of the phosphotransferase gene (*hph*) using specific primers hph-F&R to amplify an 811 bp fragment of transformants Lanes 1-4: transformants Bgl-1, Bgl-2, Bgl-3 and Bgl-4, respectively; Ma: 200 bp DNA Marker; (**B**) PCR analysis of the gene expression cassette (P*pro*A-*Bgl1*-T*trp*C) using specific primers P*proA*-F&T*trpC*-R to amplify a 4194 bp fragment of transformants. Lane 1: host strain EU7-22; Lanes 2–5: transformants Bgl-1, Bgl-2, Bgl-3 and Bgl-4. Mb: DL10 000 DNA Marker.

To investigate the resistance to glucose repression of the putative *proA* promoter, β-glucosidase gene (*Bgl1*) from *H. orientalis* was used as a reporter. The binary expression vector (pUR5750-Bgl1) was constructed, and transfered into *H. orientalis* by *Agrobacterium tumefaciens* mediated transformation (ATMT). After cultivated 3–6 days on PDA selecting plates, four putative transformants were visible. When inoculating the transformants to new plate containing 100 μg/mL hygromycin B, they all grew well. The mitotic stability of the transformants was tested by growing them on PDA plates. After successively repeating three generations, the transformants were inoculated on PDA plates containing 100 μg/mL hygromycin B. All the transformants grew well, which showed that it was mitotically stable. The four stable hygromycin B-resistant transformants were obtained and named as Bgl-1, Bgl-2, Bgl-3 and Bgl-4. PCR assay with primer hph-F&R amplified an 811 bp fragment, which suggested that the *hph* gene was successfully integrated into the genomes of the four transformants (Figure 3A). Another PCR with primer PproA-F&TtrpC-R for amplifying the 4194 bp gene expression cassette (P*pro*A-*Bgl1*-T*trp*C) fragment was performed to testify whether the putative *proA* promoter, *bgl1* gene and *trpC* terminator were synchronously integrated into the genomes. Data indicated only transformants Bgl-2 and Bgl-3 were successfully integrated into the genomes (Figure 3B, lanes 3 and 4). The host strain EU7-22 was used as the control, which no gene expression cassette fragment was amplified (Figure 3B, lane 1). The ATMT method was an effective and simple technique for fungal transformation, but ATMT mediating T-DNA integration the host genome appeared to be a random event [15,16], the truncation of the inserted T-DNA would be possible. The phenomena also occurred in *Fusarium oxysporum*[17] and *Aspergillus awamori*[18].

### 2.3. Comparison Analysis of Cellulase Production of Transformants and Host Strain EU7-22

**Figure 4:**
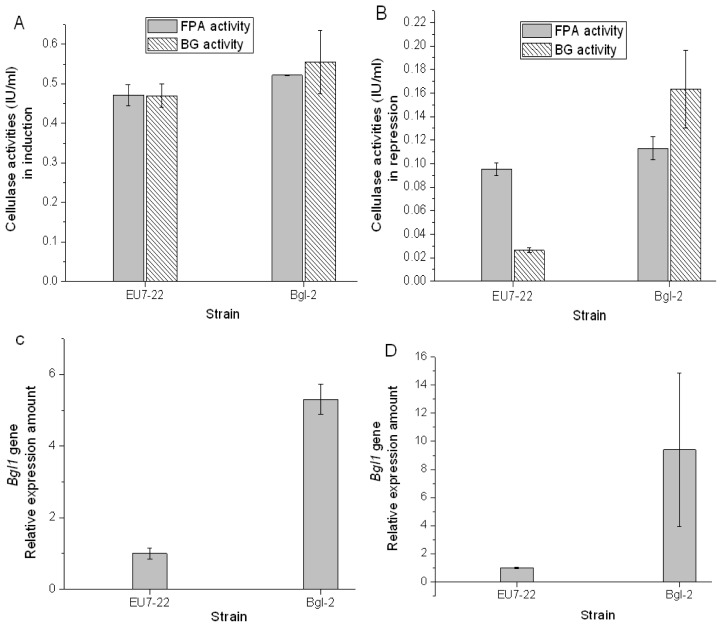
Analysis of FPA and BG activities and *bgl1* gene relative expression amount of host strain EU7-22 and transformant Bgl-2. (**A**) FPA and BG activities under induction condition; (**B**) FPA and BG activities under repression condition; (**C**) relative expression amount of *Bgl1* gene under induction condition; (**D**) relative expression amount of *Bgl1* gene under repression condition.

According to cellulase production analysis (data not shown), the transformant Bgl-2 was selected as the work strain. The pretreated *Miscanthus* cellulose was used as inducer to produce cellulase, the filter paper activity (FPA) and β-glucosidase (BG) activity of host strain EU7-22 were 0.47 IU/mL and 0.47 IU/mL, respectively; the FPA and BG activities of strain Bgl-2 were 0.52 IU/mL and 0.56 IU/mL, respectively (Figure 4A). In comparison with strain EU7-22, the FPA and BG activities of transformant Bgl-2 increased by 10.6% and 19.1% under induction conditions, respectively, which owed to the over-expression β-glucosidase gene. While a final concentration of 2% glucose was used as repressor and supplemented in the induction medium, the FPA and BG activities of host strain EU7-22 were 0.09 IU/mL and 0.02 IU/mL, respectively; the FPA and BG activities of strain Bgl-2 were 0.11 IU/mL and 0.16 IU/mL, respectively (Figure 4B). In comparison with host strain EU7-22, the FPA and BG activities of transformant Bgl-2 increased by 22.2% and 700%, respectively.

Both FPA activity and BG activity would decrease under glucose repression condition. FPA activity reflected the total activity of cellulase system. BG activity represented the enzyme activity of β-glucosidase. In comparison with the induction condition, the BG activities of host strain EU7-22 decreased 95.7% under 2% glucose repression, only 4.3% BG activity was remained, which means that the expression of *Bgl1* gene was almost been inhibited. However, the BG activity of transformant Bgl-2 only declined 71.4% under glucose repression. There are two β-glucosidase expression systems, one was driven by putative promoter *proA*, and another was driven by the original endogenous promoter of *Bgl1* gene from *H. orientalis*, which was repressed in the presence of glucose. The BG activity of strain Bgl-2 reached 0.16 IU/mL under 2% glucose repression, which equals 34.0% activity of host strain under induction condition, and 800% activity of host strain under repression condition. It suggested that the transferred *bgl1* gene expression system promoted by the putative promoter *proA* was not inhibited by 2% glucose repression. The over-expression system could express a quantity of 22%–34% β-glucosidase that produced by host strain EU7-22 under induction condition.

The transcription level of *bgl1* gene was analyzed by qRT-PCR. In the induced cellulase production, the amount of *bgl1* transcripts in strain EU7-22 was set as 1. The relative mRNA expression of *bgl1* transcripts in strain Bgl-2 increased by 5.3 fold (Figure 4C). Under glucose repression condition, the amount of *bgl1* transcripts in strain EU7-22 was also set as 1. The relative mRNA expression of *bgl1* transcripts in strain Bgl-2 increased by 9.4 fold (Fig 4D). The results showed that the expression of β-glucosidase promoted by the *proA* promoter and was not inhibited by 2% glucose. The putative promoter *proA* was an idea promoter for expressing cellulase genes under glucose repression condition.

## 3. Experimental Section

### 3.1. Microorganism Strains and Culture Conditions

The cellulase-producing strain *Hypocrea orientalis* EU7-22 was identified according to its ITS (GenBank accession no. KC751873) and tef1 sequence (GenBank accession No. KC751874), and were analysed by *Trich*OKey v. 2.0: The Molecular Barcode Program (http://www.isth.info/index.php).The strain was screened by this laboratory, and was used as a host cell for over-expression of *Bgl1* gene. It was preserved at −80 °C in a 20% glycerol solution. Before inoculation preparation, it was activated on potato dextrose agar (PDA) slants, and incubated in liquid PDA on a rotary shaker (30 °C, 34–36 h, 180 rpm), and then transferred into submerged fermentation medium with 10% (*v*/*v*) inoculation quantity (10^6^ spores mL^−1^). *Escherichia coli* DH5a was used for vector construction and propagation. The *Agrobacterium tumefaciens* AGL1 was used to mediate transformation and was grown either on Luria-Bertani (LB) broth (50 μg/mL kanamycin) or induction medium (IM) [19] supplemented with 0.2 mM acetosyringone (IMAS). Transformants were selected on PDA medium supplemented with hygromycin B (100 μg/ml), 0.2 mM cefotaxime and 20% Triton-X100.

### 3.2. Promoter proA Cloning and Analysis

**Figure 5:**
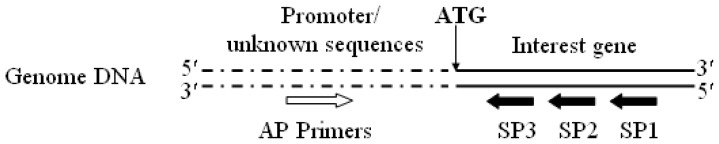
Sketch map of designed reverse primers sites for SP1, SP2 and SP3. The reverse primers (SP1, SP2 and SP3) were designed according to the interest gene (*proA*) sequence from *H. orientalis* EU7-22.

The aspartic protease could express at high level under glucose repression condition. The gene *proA* encoding for aspartic protease has been cloned, and the gene sequence was deposited at the GenBank database with accession no. JQ728540. The putative promoter *proA* of *H. orientalis* was expected as the potential enhanced promoter for expressing cellulase genes under glucose repression condition. The genome walking technology [20,21] was used to amplify the unknown region sequences based on the identified gene sequences. Genomic DNA of *H. orientalis* EU7-22 was extracted from the available mycelia according to the method of Penttilä *et al.*[22]. Specific reverse primers, SP1, SP2 and SP3 (Table 1; Figure 5), were designed according to the cloned *proA* gene sequence from *H. orientalis*. The forward primers AP1, AP2, AP3 and AP4 were supplied in the Genome Walking Kit (Takara, Japan).

The first nested PCR reaction was applied by using genomic DNA as the template, with specific primer SP1 which was paired with AP1, AP2, AP3 and AP4 in four separate reactions). The PCR condition was designed as: 94 °C for 1 min, 98 °C for 1 min, then 5 cycles of amplification (94 °C for 30 s, 62 °C for 1 min, 72 °C for 2 min), 94 °C for 30 s, 25 °C for 3 min, 72 °C for 2 min, then 15 cycles of amplification (94 °C for 30 s, 62 °C for 1 min, 72 °C for 2 min, 94 °C for 30 s, 62 °C for 1 min, 72 °C for 2 min, 94 °C for 30 s, 44 °C for 1 min, 72 °C for 2 min), and finally 72 °C for 10 min. The PCR products were shown in Figure 1 (lanes 1–4).

The second nested PCR was carried out by using the first nested PCR products as templates, with specific primer SP2 and primer AP1, AP2, AP3 and AP4, respectively. The PCR condition was designed as: 15 cycles of amplification (94 °C for 30 s, 62 °C for 1 min, 72 °C for 2 min, 94 °C for 30 s, 62 °C for 1 min, 72 °C for 2 min, 94 °C for 30 s, 44 °C for 1 min, 72 °C for 2 min), then 72 °C for 10 min. The PCR products were shown in Figure 1 (lanes 5–8).

The third nested PCR was carried out by using the second nested PCR products as templates, with specific primer SP3 and primer AP1, AP2, AP3 and AP4, respectively. The PCR condition was designed as: 15 cycles of amplification (94 °C for 30 s, 62 °C for 1 min, 72 °C for 2 min, 94 °C for 30 s, 62 °C for 1 min, 72 °C for 2 min, 94 °C for 30 s, 44 °C for 1 min, 72 °C for 2 min), then 72 °C for 10 min. The PCR products were shown in Figure 1 (lanes 9–12). The third round PCR products were purified, directly sequenced and validated. The resulting putative promoter sequence was then analyzed.

### 3.3. Construction of Binary Expression Vectors pUR5750-Bgl1

**Figure 6:**
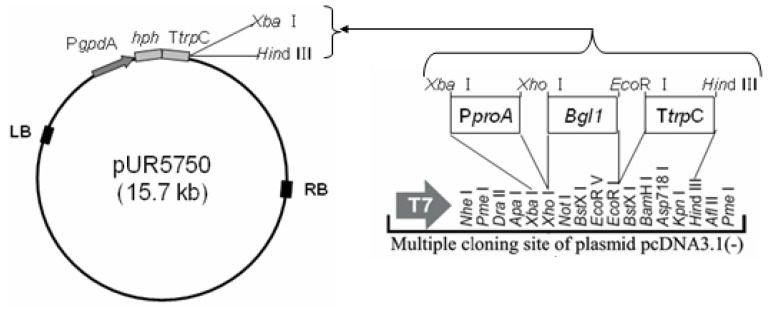
Schematic illustration of the binary expression vectors pUR5750-Bgl1 construction.

The putative *proA* promoter (1200 bp) was amplified by PCR with primer PproA-F&R (contain *Xba* I and *Xho* I site). The *bgl1* gene (2235 bp) was amplified from *H. orientalis* mRNA by RT-PCR with primer Bgl1-F&R (contain *Xho* I and *Eco*R I site), The *trpC* terminator (740bp) was amplified from plasmid pUR5750 with primer TtrpC-F&R (contain *Eco*R I and *Hin*d III site). The plasmid pUR5750 was used as a backbone to construct the binary expression vector, and a hygromycin B resistant gene (*hph*) was adopted as a selecting marker for screening *H. orientalis* transformats. The dual expression vector pUR5750-Bgl1 was built by means of the intermediate plasmid pcDNA3.1 (−) (Figure 6). The primers used in this study were listed in Table 1.

### 3.4. Agrobacterium-Tumefaciens-Mediated-Transformation (ATMT) in *H. orientalis* EU7-22

*A.tumefaciens* AGL-1, containing a binary vector (pUR5750-Bgl1), was grown at 28 °C for 2 days in LB supplemented with kanamycin (50 μg/mL). The AGL-1 cells were diluted to (optical density) OD_600_ = 0.15 in induction medium (IMAS), and were grown for an additional 8 h until an optical density at 660 nm of 0.4 before mixing them with an equal volume of a conidial suspension from strain EU7-22 (1 × 10^6^ conidia per mL). This mix (200 μL per plate) was spread onto an IMAS agar plate for co-cultivation (same as IMAS except containing 5 mM glucose instead of 10 mM glucose). Following incubation at 25 °C for 48 h, then place the PDA medium supplemented with hygromycin B (100 μg/mL), 0.2 mM cefotaxime and 20% Triton-X100 onto IMAS agar plate, sequentially incubated at 30 °C for 3–6 days, and the visible putative transformants were chosen.

### 3.5. Transformant Stability and Molecular Analysis of the Transformants

The putative visible transformants was firstly picked up and transferred to a PDA agar plate containing hygromycin B. The transformants were then cultured on PDA agar plate without hygromycin B by three times. Then the monoconidial cultures were transferred to PDA plates containing 100 μg/mL hygromycin B for determining the stability of the transformant. Genomic DNA of transformant was extracted from all available mycelia according to the method of Penttilä *et al.*[22]. The gene fragments were analysed by PCR amplification using primer hph-F&R (for 811 bp) and PproA-F &TtrpC-R (for 4194 bp).

### 3.6. Enzymatic Analysis

Experiments were conducted in the 250-mL Erlenmeyer flasks. The induction medium (pH 5.2) for cellulase production was 2% pretreated *Miscanthus* cellulose (the dry *Miscanthus* straw was crashed into powder, extracted with 2% NaOH and 1% H_2_O_2_ for 2h at 60 °C, then extracted with 2% NaOH for 2 h at 100 °C, wash to neutral and drying), 1% wheat bran, 0.5% peptone, 0.05% CaCl_2_, 0.05% MgSO_4_, 0.4% Tween 80 and 2.5 g/L KH_2_PO_4_. The repression medium was supplemented glucose (final concentration 2%) to the induction medium to act as a repressor. They were incubated on a rotary shaker (30 °C, 4 days, 180 rpm). Crude enzyme was firstly centrifugated (6000 rpm, 10 min) to remove the cells and solid material. The enzyme activity of the supernatant was then determined. Filter paper activity (FPA) was measured as described by Ghose [23]. A standard curve of d-glucose was used as a reference. One unit of enzyme activity was defined as the amount of enzyme required to liberate 1 μM of reducing sugar per minute, and expressed as IU mL^−1^. β-glucosidase (BG) activity were assayed as described by Saha [24]. A standard curve of *p*-nitrophenol (*p*NP) was used as a reference. One unit of enzyme activity was defined as the amount of enzyme that released 1 μmol *p*NP per minute in the reaction, and expressed as IU mL^−1^. The protein concentration of crude enzyme was measured with a Bradford protein assay kit (Sangon Biotech Co. Ltd., Shanghai, China).

### 3.7. Transcription Analysis

Approximately 200 mg of mycelium was ground to a fine powder under liquid nitrogen and transferred to a 50 mL Corning tube on ice. RNA was extracted from the mycelium of *H. orientalis* and transformants by Trizol reagent (Takara). The quality of the extracted total RNA was identified by agarose gel electrophoresis and the concentration of mRNA was measured by spectroscopy. Reverse transcription was carried out using the PrimeScript^®^ RT reagent Kit (Takara). Relative expression levels of *bgl1* were calculated in comparison with the expression of 18S rRNA gene by Real-Time PCR (ABI StepOnePlus) with primers BGLYG-F&R, 18s-F&R.

## 4. Conclusions

A novel putative promoter *proA,* which was resistant to glucose repression, was successfully isolated by genome walking. The binary vector pUR5750-Bgl1 for expressing *bgl1* gene with promoter *proA* was constructed and transferred into *H. orientalis* via ATMT. Under the induction condition, the FPA and BG activity of transformant strain Bgl-2 increased by 10.6% and 19.1% compare with the host strain EU7-22, respectively. Under the repression condition with a final concentration of 2% glucose, the FPA and BG activity of strain Bgl-2 increased by 22.2% and 700% compare to host strain EU7-22, respectively. The *bgl1* gene expression system promoted by promoter *proA* in strain Bgl-2 could highly express β-glucosidase under both induction and glucose repression. The over-expression system could express a quantity of 22%–34% β-glucosidase, which produced by host strain EU7-22 under the induction condition.

## Figures and Tables

**Table 1:** Primers used in this study for polymerase chain reaction.

Primer name	Primer sequence (5′–3′)
SP1-R^b^	AGACGGAGGTGATGTACTCCGAATC
SP2-R^b^	CTTCTTGGCCTTGAAGAGAGCAGTAG
SP3-R^b^	CGAGGAAGGAAACGAGAAAAGCTC
M13-F^a^	CGCCAGGGTTTTCCCAGTCACGAC
M13-R^b^	GAGCGGATAACAATTTCACACAGG
PproA-F^a^	CTAGTCTAGAAGTAGCAAAGGCTAACGGATTAG
PproA-R^b^	CCGCTCGAGCTTGAATCTCGGAGAGGGCTG
Bgl1-F^a^	CCGCTCGAGATGCGTTACCGAACAGCAG
Bgl1-R^b^	CCGGAATTCCTACGCTACCGACAGAGTGCT
TtrpC-F^a^	CCGGAATTCAGTAGATGCCGACCGCG
TtrpC-R^b^	CCCAAGCTTAAGAAGGATTACCTCTAAACAAG
hph-F^a^	CGACAGCGTCTCCGACCTGA
hph-R^b^	CGCCCAAGCTGCATCATCGAA
BGLYG-F^a^	ATCACCTACCCGCCTTCA
BGLYG-R^b^	TCTCGTCGTCGGATGTTG
18s-F^a^	AGGCGCGCAAATTACCCAATCC
18s-R^b^	GCCCTCCAATTGTTCCTCGTTAAG

F^a^: forward primer; R^b^: reverse primer.
